# Influenza-related hospitalization of adults associated with low census tract socioeconomic status and female sex in New Haven County, Connecticut, 2007-2011

**DOI:** 10.1111/irv.12231

**Published:** 2014-01-02

**Authors:** Karman Tam, Kimberly Yousey-Hindes, James L Hadler

**Affiliations:** a Connecticut Emerging Infections Program, Yale School of Public HealthNew Haven, CT, USA; b Office of Infectious Disease Services, Arizona Department of Health ServicesPhoenix, AZ, USA

**Keywords:** Disparities, female, hospitalization, Influenza, sex, socioeconomic status

## Abstract

**Objectives:**

To help guide universal influenza vaccination efforts in the United States, it is important to know which demographic groups are currently at highest risk of costly complications of influenza infection. Few studies have examined the relationship between hospitalization with influenza and either socioeconomic status (SES) or sex. We examined associations between census tract-level SES and sex and incidence of influenza-related hospitalizations among adults.

**Design:**

Descriptive analysis of data collected by active population-based surveillance for persons >18 years old hospitalized with laboratory confirmed influenza during the 2007–2008 through 2010–2011 influenza seasons. Case residential addresses were geocoded and linked to data from the 2006–2010 American Community Survey to obtain census-tract level (neighborhood) SES measures. Census-tract level SES variables included measures of poverty, education, crowding, primary language, and median income. Four levels were created for each.

**Setting:**

New Haven, County, Connecticut.

**Sample:**

Entire New Haven County population >18 years old.

**Main Outcome Measures:**

Age-adjusted incidence of influenza hospitalizations and relative risk by sex and by each of five SES measures.

**Results:**

Crude and age-adjusted incidence progressively increased with decreasing neighborhood SES for each measure both overall and for each influenza season. Female incidence was higher than male for each age group, and female age-adjusted incidence was higher for each SES level and influenza season.

**Conclusions:**

Female sex and lower neighborhood SES were independently and consistently associated with higher incidence of hospitalization of adults with influenza. If this is more broadly the case, these findings have implications for future influenza vaccination efforts. Analysis using census tract SES measures can provide additional perspective on health disparities.

## Introduction

In the United States, a yearly average of 5–20 percent of the population contracts influenza and over 200 000 are hospitalized for influenza-related illnesses.^1^,^2^ The estimated number of resulting deaths each year has ranged from 3000 to 49 000 depending on the influenza strains circulating.^3^ Young children, the elderly, and individuals with certain chronic medical conditions are at particularly high risk of developing influenza-related complications including hospitalization and death.^4^ To reduce the morbidity, mortality, and economic loss associated with influenza, vaccination is recommended for all individuals 6 months of age and older, especially for persons at high risk.^4^

In addition to persons at high risk, some demographic groups in the population may suffer disproportionately from influenza. While most disease surveillance systems based on laboratory and provider reporting capture age, sex, and race/ethnicity, information related to socioeconomic status (SES) is rarely collected. This results in inability to identify and address issues related to socioeconomic disparities and health.^5^ Studies linking surveillance data with data from the U.S. Census to determine disease incidence by neighborhood SES have been shown to be of significant value because a person's health is often directly influenced by the conditions of one's neighborhood.^5^ In Connecticut, a previous study found influenza hospitalization in children to be strongly associated with higher neighborhood poverty levels and with household crowding in each of seven consecutive influenza seasons.^6^ A case–control study of pandemic H1N1 in New York City October 2009–February 2010 found an association for both children and adults between being hospitalized with influenza and low SES status defined by zipcode-level poverty.^7^ However, a population-based analysis in adults to confirm and examine the consistency of this finding over time has not been done.

In a national 10-site Emerging Infections Program population-based study of adults hospitalized with influenza from 2005 to 2008, women were found to account for more than 56% of 5,054 adults hospitalized with influenza. The different percentages of women and men could not be accounted for by hospitalization of pregnant women.^8^ This finding has not been examined to determine whether it is consistent across race/ethnic and SES groups or if it can be explained by differences in other underlying conditions or vaccination.

We used data from the Connecticut Emerging Infections Program's (CT-EIP) influenza-associated hospitalization surveillance system to (i) determine whether incidence of hospitalization with confirmed influenza among adults was associated with neighborhood SES, (ii) examine whether other measures of SES describe disparities as well as the recommended measure for surveillance, percentage living below the federal poverty level^5^, and (iii) determine whether adult female incidence is higher than male and to which female demographic groups this might apply.

## Methods

### Surveillance data

The CT-EIP has been conducting population-based surveillance for hospitalized, laboratory-confirmed influenza among all adults in New Haven County (population 845 244 in 2010) since October 2007. Positive laboratory tests for influenza are a reportable condition in Connecticut. We followed up all positive reports from New Haven County residents with hospitals to determine whether the patient had been hospitalized. Through chart reviews and interviews with healthcare providers and with patients or their proxies, the following data were collected using a standardized case report form for each hospitalized case from 2007 to 2011: demographic information (age, sex, race/ethnicity), residential street address, influenza test results, underlying medical conditions identified by the Advisory Committee on Immunization Practices (ACIP) that increase the risk for influenza complications (e.g., pregnancy, asthma, cardiovascular disease, chronic respiratory disease, chronic metabolic disease, renal failure, immunosuppressive condition), and vaccination history. If vaccination history was not specified in the medical record, efforts were made to obtain it from first, the primary care provider and, if there is no clear record, the patient.

### Census data

We followed the process used by the Public Health Disparities Geocoding Project (PHDGP), a project exploring use of area-based SES data in public health surveillance systems to describe and monitor health disparities.^9^ Census tract-level SES data were obtained from the U.S. Census Bureau's 2006–2010 American Community Survey (ACS).^10^ From the 2006 to 2010 ACS, the variables percentage living below the federal poverty level, with less than a high school education, living in rooms with >1 occupant, living in non-English speaking households, and median household income were selected as SES characteristics to be examined at the census tract level.

For percentage living below the federal poverty level, four categories were used (0–4·9%, 5–9·9%, 10–19·9%, and ≥20%) based on recommendations of the PHDGP and as used previously in Connecticut.^9^,^11^,^12^^.^

Percentage not having obtained a high school diploma was based on responses among those ≥25 years old.^10^ Four levels were created for this category (0–14·9%, 15–24·9%, 25–39·9%, and 40% and higher), consistent with those used in another EIP study.^13^

We used the percentage of households in a census tract with more than one occupant per room to indicate crowding.^10^ Four levels were created (0–0·9%, 1·0–2·9%, 3·0–4·9%, and ≥5%) as used previously.^6^

Four levels were developed for percentage of households with persons who do not speak English (0–3·9%, 4–7·9%, 8–11·9%, 12% and higher), beginning with the median level (3·9%).

Median household income was broken into four levels (<$25 000, $25 000–49 999, $50 000–74 999, and $75 000 and above) based on previous PHDGP studies.^5^

Overall adult and group-specific population estimates for New Haven County were obtained from the 2010 Census.

### Geocoding

New Haven County has 185 census tracts. Using arcmap/arcgis software version 9.2 (ESRI, Redlands, CA, USA), each patient's home address was geocoded to determine the census tract of residency. Efforts were made to geocode manually all street addresses that were not able to be geocoded using the software.

### Statistical analysis

The incidence for each census tract-level socioeconomic characteristic stratum was calculated by dividing the total number of cases in all census tracts with the given characteristic by the total number of adults residing in them. Average annual incidence was calculated by dividing the overall incidence by four, the number of influenza seasons examined. The spring 2009 H1N1 outbreak was included as part of the 2008–2009 influenza season. Chi-squared tests for trend were used to determine whether a significant trend existed within each neighborhood SES measure. Because age was a strong predictor of hospitalization with influenza, we calculated age-adjusted rates by SES level and by sex, using the three age groups examined and standardizing to the 2010 New Haven County population.

We analyzed the percentage of cases who had at least one underlying condition that is an indication for influenza vaccination in adults and who had been vaccinated against influenza earlier in the same season they were hospitalized, by age, by sex, and by each census tract poverty level and used the chi-squared test for trend to test for association with census tract poverty level. For calculations excluding pregnant women from the denominator, we assumed each woman was pregnant for 9 months and reduced the number of women in the denominator for each year by 0·75 times the number of live births that year. All statistical analyses were performed using sas version 9.2 (SAS Institute Inc, Cary, NC, USA) or EpiInfo version 3.3.2 (Centers for Disease Control and Prevention, Atlanta, GA, USA).

## Results

During the four influenza seasons, a total of 1137 adults were hospitalized with laboratory-confirmed influenza in New Haven County (range by year, 200 in 2008–2009 to 364 in 2007–2008). Of these, 1094 (96·4%) were successfully geocoded and included in the analysis. Forty-three addresses could not be geocoded because they were P.O. Boxes, incomplete or invalid. Residents of long-term care facilities were geocoded to the address of their facility.

### Demographic distribution

The average annual incidence of influenza-related hospitalizations differed by age, gender, and race/ethnicity (Table 1). Increasing age was strongly associated with higher incidence with incidence among those ≥65 years more than four times higher than among those 18–49 (relative risk (RR) 4·58, 95% confidence interval (CI) 3·99–5·25). By sex, the overall incidence among women was 1·48 times higher than among men and was still 1·35 times higher after age adjustment (aRR 1·35, 95% CI 1·20–1·53). Higher rates in women compared to men were seen among each age group (range of RR, 1·76 (95% CI 1·41–2·20) times higher among 18- to 49-year-olds to 1·19 (95% CI 0·99–1·42) among ≥65-year-olds) and for each influenza season including the 2009–2010 H1N1pdm season (age-adjusted ranged from 1·88 in 2008–2009 to 1·49 during 2009–2010 to 1·34 in 2007–2008 to 1·07 in 2010–2011). By race/ethnicity, compared to non-Hispanic whites, incidence in non-Hispanic blacks was 2·19 times higher and in Hispanics was 1·46 times higher. Age adjustment further increased race/ethnic disparities (aRR 2·77, 95% CI 2·39–3·21 for blacks; aRR 2·20, 95% CI 1·89–2·56 for Hispanics). When examined by sex, the age-adjusted female-to-male incidence ratio was >1 for each race/ethnic group: 1·11 (95% CI 0·95–1·31) for non-Hispanic whites, 1·59 (95% CI 1·22–2·06) for non-Hispanic blacks, and 2·57 (95% CI 1·91–3·46) for Hispanics.

**Table 1:** Average annual incidence and crude and age-adjusted relative risk of influenza-associated hospitalizations among adults by age, by sex, and by race/ethnicity, New Haven County, CT, 2007–2011 (*N* = 1094)

	Population*	Number (%) of cases	Crude average annual incidence**	Relative risk and 95% CI	*P*-value	Age-adjusted relative risk and 95% CI
Age category						
18–49	374 115	340 (31·1)	22·72	Reference		Not applicable
50–64	171 416	238 (21·8)	34·71	1·53 (1·29–1·80)	<0·001 (trend)	Not applicable
65+	123 972	516 (47·26)	104·06	4·58 (3·99–5·25)		Not applicable
Sex						
Male	316 520	413 (37·8)	32·62	Reference	–	Reference
Female	352 983	681 (62·2)	48·23	1·48 (1·31–1·67)	<0·001	1·35 (1·20–1·53)
Race/ethnicity***						
White (non-Hispanic)	478 657	673 (61·5)	35·15	Reference	–	Reference
Black (non-Hispanic)	73 409	226 (20·7)	76·97	2·19 (1·88–2·55)	<0·001	2·77 (2·39–3·21)
Hispanic	84 123	173 (15·8)	51·41	1·46 (1·24–1·73)	<0·001	2·20 (1·89–2·56)

* Population estimates obtained through the 2010 Census.

** Overall incidence was calculated as the number of cases within the category dividing by the total population within the category. Average annual incidence was then calculated for the 4 study season as per 100 000 person-years.

*** Twenty-two (2·0%) cases were either in other race/ethnicity categories or the data on race/ethnicity were missing.

### Neighborhood socioeconomic condition and influenza hospitalization

There were notable trends in the crude and age-adjusted average annual incidence of influenza-related hospitalizations by each neighborhood SES characteristic (Table 2). The incidence increased as the percent of persons living below poverty in a census tract increased, as the percent of persons in a census tract with no high school diploma increased, as the percent of crowded households in a census tract increased, as the percent of non-English speaking households in a census tract increased, and as median income in the census tract decreased. These trends were present in each influenza season including the 2009-10 H1N1pdm season (Table S1).

**Table 2:** Average annual age-adjusted incidence of influenza-associated hospitalizations among adults by neighborhood SES characteristic and by race/ethnicity, New Haven County, CT, 2007–2011 (*N* = 1094)

Neighborhood characteristic	Number (%) of cases	Overall age-adjusted** incidence*	Non-Hispanic white Age-adjusted incidence*	Non-Hispanic black Age-adjusted incidence*	Hispanic age-adjusted incidence*
Below poverty***					
Low (0–4·9%)	340	26·45	24·84	45·36	26·43
Medium–low (5–9·9%)	180	39·72	34·55	77·30	68·83
Medium–high (10–19·9%)	297	59·94	47·67	80·11	53·07
High (≥20%)	277	78·20	45·30	106·48	91·73
No high school diploma					
Low (0–14·9%)	645	34·35	29·39	76·36	50·04
Medium–low (15–24·9%)	267	54·86	40·19	79·37	57·57
Medium–high (25–39·9%)	138	85·32	51·80	108·93	107·15
High (≥40%)	44	78·35	58·00	105·76	72·24
crowding					
Low (0–0·09%)	402	29·26	26·54	50·80	35·93
Medium–low 1–2·9%	288	42·01	34·21	71·04	55·75
Medium–high (3–4·9%)	193	71·87	51·22	101·50	69·33
High (≥5%)	211	80·96	53·65	114·53	103·81
Non-English speaking households					
Low (0–3·9%)	479	31·77	28·02	63·03	33·62
Medium–low (4–7·9%)	335	43·76	33·89	79·01	56·12
Medium–high (8–11·9%)	113	82·72	63·11	100·25	74·28
High (≥12%)	167	100·83	66·08	132·79	113·74
Median income					
High (≥$75 000)	57	23·62	23·54	25·11	26·19
Medium–high ($50 000–$74 999)	463	36·14	32·25	65·57	44·40
Medium–low ($25 000–$49 999)	356	75·51	48·72	100·64	87·66
Low ($0–24 999)	218	77·35	63·35	87·48	66·24

* *P* < 0·01 by chi-squared test for trend for each category in the column except for blacks with no high school diploma (*P* = 0·03).

** Age-adjusted by three age groups: 18–49 years, 50–64 years, and ≥65 years, standardized to the 2010 New Haven County, Connecticut, adult population.

*** Poverty defined as the percentage of persons living below the federal poverty level in a census tract per the 2006–2010 American Community Survey.^12^

Age-adjusted rates by race/ethnicity for the percentage living in poverty also revealed statistically significant trends of increasing incidence by increasing poverty level within each race/ethnic group (Table 2). Age-adjusted rates were highest among non-Hispanic blacks and lowest among non-Hispanic whites for each level of each SES variable.

Age-adjusted rates by sex showed females had higher rates than males for each census tract poverty level, with higher relative rates the higher the poverty level (1·13 for low poverty, 1·23 for medium–low poverty, 1·36 for medium–high poverty, and 1·76 for high poverty, *P* < 0·001, chi-squared test for trend).

### Underlying conditions

The percentage of cases with underlying conditions was examined to determine whether there were differences between groups that could explain the differences in incidence by neighborhood poverty level or by sex.

There were no differences in the percentages with any underlying condition by census tract poverty level (Table 3). However, asthma was increasingly prevalent among cases with progressively higher census tract poverty status (16·4% in low poverty versus 37·7% in high poverty), while cardiovascular disease was more prevalent as case census tract poverty status decreased (43·3% high poverty versus 57·3% low poverty) (*P* < 0·001 for each).

**Table 3:** Number and percentage of adults with influenza-associated hospitalization with specified underlying conditions*** by age group and sex and by census tract poverty level, New Haven County, 2007–2011

Characteristic	Total cases excluding pregnant women	Any underlying condition (excluding pregnancy)	Cardiovascular disease	Chronic lung disease	Asthma	Pregnancy
		*N* (%)	*N* (%)	*N* (%)	*N* (%)	*N* (%)
All female	633	571 (90·2)	328 (51·8)	159 (25·1)	175 (27·6*)	48
All males	413	360 (87·2)	219 (53·0)	84 (20·3)	69 (16·7)	NA
Female 18–49	172	138 (80·2)	29 (16·9)	24 (14·0	85 (49·4*)	48
Male 18–49	120	97 (80·8)	23 (19·2)	12 (10·0)	44 (36·7)	NA
Female 50–64	137	124 (90·5)	62 (45·3)	37 (27·0)	49 (35·8*)	0
Male 50–64	101	83 (82·2)	47 (46·5)	18 (17·8)	14 (13·9)	NA
Female 65+	324	309 (95·4)	237 (73·1)	98 (30·2)	41 (12·7*)	NA
Male 65+	192	180 (93·8)	149 (77·6)	54 (28·1)	11 (5·7)	NA
Below poverty†						
Low (0–4·9%)	335	291 (86·9)	192 (57·3)	74 (22·1)	55 (16·4)	5
Medium–low (5–9·9%)	177	159 (89·8)	97 (54·8)	55 (31·1)	26 (14·7)	3
Medium–high (10–19·9%)	282	253 (89·7)	149 (52·8)	61 (21·6)	68 (24·1)	15
High (≥20%)	252	228 (90·5)	109 (43·3**)	53 (21·0)	95 (37·7**)	25

* *P* < 0·05 for percentage female versus percentage male.

** *P* < 0·01 for percentage highest versus percentage lowest poverty and for trend across all four groups.

*** Excluding pregnant women.

† Poverty defined as the percentage of persons living below the federal poverty level in a census tract per the 2006–2010 American Community Survey.^9^

When examined by sex, there were few statistically significant differences between females and males except for one: females overall and in each age group were more likely to have asthma (overall, 27·6% versus 16·7%, *P* < 0·001) (Table 3).

Of all female admissions, 7·0% (48) were in pregnant women. When pregnant women were removed from both the numerator and denominator, females still had a significantly higher age-adjusted incidence rate compared to males (RR = 1·26, 95% CI 1·11–1·42), including those 18–49 years old (RR = 1·40, 95% CI 1·11–1·76).

### Vaccination

Among cases with available vaccination information, the percentage who were vaccinated was also examined to determine whether there were differences between groups that could explain the differences in incidence by neighborhood poverty level or by sex. Data on influenza vaccination were available for 1035 (94·6%). There were no differences in the percentages for whom vaccination status information was present by poverty category or age, but females were slightly more likely than males to have information (95·7% versus 92·7%, *P* = 0·03). Of those with information, 513 (49·6%) had been vaccinated earlier in the relevant influenza season. Vaccination was strongly associated with age (30·6% for 18- to 49-year-olds, 39·9% for 50- to 64-year-olds, and 66·1% for ≥65-year-olds, *P* < 0·001, chi-squared test for trend) and poverty level (55·6% for low, 50·9% for medium–low, 49·7% for medium–high, and 41·2% for high) (Table 4).

**Table 4:** Number and percentage of adults with influenza-associated hospitalization who were vaccinated against influenza, by age, sex, and poverty level, New Haven County, CT, 2007–2011 (*N* = 1023)

	Number of cases with vaccine data (*n* = 1035)^a^	Number (%) vaccinated (*n* = 513)	*P*-value*
Age group			
18–49 years	317	97 (30·6)	<0·001
50–64 years	223	89 (39·9)	
65+ years	495	327 (66·1)	
Sex			
Female	652	320 (49·1)	0·68
Male	383	193 (50·4)	
Below poverty			
Low (0–4·9%)	322	179 (55·6)	<0·001
Medium–low (5–9·9%)	167	85 (50·9)	
Medium–high (10–19·9%)	286	142 (49·7)	
High (≥20%)	260	107 (41·2)	

* Chi-squared test for trend for age group and for below poverty.

## Discussion

Our study of four recent influenza seasons revealed important disparities in the incidence of influenza-related hospitalizations by neighborhood SES and by sex. For each of five different area-based SES measures, a significant trend of increasing incidence of influenza-associated hospitalizations was found with decreasing SES status. This trend was also found during each influenza season and within each major race/ethnic group. In addition, we found that blacks and Hispanics had substantially higher rates of hospitalization than whites within each SES group, that the influenza hospitalization rate was higher with increasing age and that females had consistently higher rates than males in all groups defined by age, SES level, and race/ethnicity. This is the first time to our knowledge that a population-based study of the relationship between hospitalization with influenza in adults and a number of area-based SES measures has been performed on data collected across more than one influenza season. The SES findings complement those we previously reported on pediatric hospitalization using 2000 census data,^6^ extending them to adults. While the finding that females have higher rates of influenza-associated hospitalizations than males is not entirely new, it has not previously been examined to determine its consistency over time or within groups defined by age, SES, and race/ethnicity.

Adults residing in neighborhoods of lower SES may be at increased risk for influenza for several possible reasons. First, they may have increased exposure to influenza. Household crowding, a direct measure of potential domestic and neighborhood exposure, was strongly associated with increased incidence. In addition, adults are often exposed to influenza through children.^14^–^16^ Our previous study demonstrated twofold to eightfold higher rates by year of hospitalization between high- and low-poverty neighborhoods in children. To the extent hospitalization reflects influenza incidence, this could be a major likely contributing explanation for the higher rates by year seen in adults residing in the same catchment area.^6^ Second, residents from neighborhoods of lower SES are less likely to have health insurance and routine medical care.^17^ As a result, they are less likely to be immunized and underlying conditions such as asthma are less likely to be controlled.^18^ In our study, relative vaccination rates among cases, a potential surrogate for relative vaccination rates in the community, were progressively lower with decreasing SES. Third, studies have repeatedly demonstrated that neighborhoods of lower SES tend to have poorer household quality, higher rates of pest infiltration (e.g., cockroaches, mice, and rats), and resulting higher rates of asthma.^19^,^20^ Influenza exacerbates poorly controlled asthma and can lead to hospitalization. Cases in the lower SES strata had 1·5- to 2·3-fold higher asthma prevalence than those in the highest SES stratum.

We explored the use of five somewhat different census tract-level SES measures in this study. A key barrier to routinely using area-based SES measures for data analysis and comparison is lack of a standard agreed upon measure. Krieger *et al*.^5^ after exhaustive study recommended use of census tract-level percentage of residents living below the federal poverty level as a standard, understandable index of SES. Our analysis supports use of this measure as it described differences between lowest and highest SES levels in influenza hospitalization incidence and gradients in between as well as crowding and better than the other measures.

We found rates by sex in adults of all ages and in all SES groups to be consistently higher in women. Relative rates were particularly high in women 18–49 years, even after excluding those who were pregnant, and in women in lower SES groups. Assuming this finding is real and not an artifact of relative care seeking or bias in admitting ill women more readily than men, there are likely several contributing factors: higher risk of exposure to influenza and higher underlying rates of asthma in women than men. Mothers usually spend more time with their children than fathers and poorer women tend to have more children than less poor ones.^21^ When children are ill, women are more likely to stay home to care for sick children than men.^22^,^23^ A recent study of household transmission of pandemic influenza in New York City found that women in households with an ill child had a much higher risk than men in the same households.^16^ In addition, women and persons living in poverty have a higher prevalence of asthma.^20^ We found female cases in all age groups to have a higher percentage with asthma than males.

Our findings have potential implications for vaccination efforts in Connecticut and potentially other US jurisdictions if the findings can be replicated elsewhere. Currently, the ACIP recommends that all persons be vaccinated annually, with special attention to those with underlying conditions and older persons. However, at least in Connecticut, there are substantial disparities in risk of hospitalization with influenza for those in low SES neighborhoods and for younger women, and fewer of the cases and, presumably, the population in these groups are vaccinated. To reduce these disparities and the burden of hospitalization, systematic efforts are needed to achieve higher vaccination rates in low SES neighborhoods, particularly among younger women with emphasis on those with asthma and other underlying conditions, including pregnancy.

## Limitations

There are important limitations to consider in this analysis. First, neighborhood SES data were obtained from the 2006 to 2010 ACS. The ACS only includes information from a sample of the U.S. population. This could result in some misclassification of census tract SES level. Misclassification would tend to make SES differences smaller that they really were. Second, the analysis was based on neighborhood SES data, not individual SES data. Therefore, assumptions made at the neighborhood level may not apply to every resident living in the neighborhood. The intention was to examine the important effects that neighborhoods have on health rather than to use neighborhood SES as a proxy for individual SES. Third, there were important assumptions underlying analysis of differences in underlying medical conditions and vaccination levels. It was assumed that all underlying medical conditions had equal risk of causing influenza infection to be severe enough to result in hospitalization and that the probability of vaccination “failure” was equal by sex and SES group. To the extent that these assumptions were not true, some of the differences in incidence by SES and by sex may have been explainable by these factors. Finally, this analysis is limited to data collected from a single county in Connecticut. It is unknown the extent to which these findings can be generalized to other parts of the United States.

## Conclusion

Analysis of surveillance data using census tract-level SES provides a different perspective than analysis solely by race/ethnicity. Adults residing in neighborhoods of lower SES and females, regardless of census tract-level SES, were more likely to be hospitalized with influenza than other adults. Analysis of surveillance data from other areas of the United States is needed to confirm these findings and their implication: that vaccination efforts in the future should include enhanced efforts to improve vaccination levels in these groups, particularly in those with underlying conditions. A standard census tract-level SES variable is needed to better describe disparities nationally and across states: Strong consideration should be given to the percentage of the population living below the federal poverty level.

## Supporting Information

**Table S1.** Annual age-adjusted incidence of influenza-associated hospitalizations among adults by neighborhood SES characteristic, New Haven County, CT, 2007-2011 (*N* = 1094).
