# Supplementary material for: Influenza-related hospitalization of adults associated with low census tract socioeconomic status and female sex in New Haven County, Connecticut, 2007-2011
Source: Influenza Other Respir Viruses. 2014 Jan 2;8(3):274–81. doi: 10.1111/irv.12231 (PMC4181475; doi:10.1111/irv.12231)
Supplement: Supplementary file 1 — Table S1. Annual age-adjusted incidence of influenza-associated hospitalizations among adults by neighborhood SES characteristic, New Haven County, CT, 2007-2011 (N = 1094). [file irv0008-0274-SD1.docx]

Supplemental Table. Annual age-adjusted incidence of influenza-associated hospitalizations among adults by neighborhood SES characteristic, New Haven County, CT, 2007-2011 (N=1,094)

| Neighborhood characteristic | 2007-08 | | 2008-09 | | 2009-10 | | 2010-11 | |
| --- | --- | --- | --- | --- | --- | --- | --- | --- |
|  | N | I^a^ | N | I^a^ | N | I^a^ | N | I^a^ |
| Below Poverty^b^ |  |  |  |  |  |  |  |  |
| Low (0-4.9%) | 129 | 36.7 | 49 | 15.8 | 70 | 23.4 | 102 | 31.6 |
| Medium-low (5-9.9%) | 58 | 51.0 | 33 | 29.7 | 32 | 30.1 | 57 | 51.3 |
| Medium-high (10-19.9%) | 108 | 91.5 | 51 | 40.9 | 48 | 37.4 | 90 | 74.2 |
| High (>20%) | 79 | 97.8 | 67 | 68.7 | 63 | 69.8 | 68 | 82.5 |
| No high school diploma |  |  |  |  |  |  |  |  |
| Low (0-14.9%) | 232 | 49.2 | 102 | 22.5 | 123 | 27.8 | 187 | 40.2 |
| Medium-low (15-24.9%) | 79 | 69.5 | 55 | 44.6 | 45 | 35.5 | 88 | 74.0 |
| Medium-high (25-39.9%) | 41 | 93.0 | 30 | 66.8 | 36 | 74.2 | 31 | 83.2 |
| High (>40%) | 12 | 88.4 | 13 | 94.0 | 9 | 67.7 | 10 | 69.2 |
| Crowding |  |  |  |  |  |  |  |  |
| Low (0-0.09%) | 151 | 43.8 | 48 | 14.5 | 74 | 23.0 | 129 | 37.6 |
| Medium-low 1-2.9% | 84 | 50.1 | 66 | 39.2 | 51 | 30.0 | 87 | 51.7 |
| Medium-high (3-4.9%) | 76 | 121.2 | 38 | 54.9 | 41 | 59.5 | 38 | 57.2 |
| High (>5%) | 53 | 90.2 | 48 | 70.3 | 47 | 68.0 | 63 | 101.5 |
| Non-English Speaking Households |  |  |  |  |  |  |  |  |
| Low (0-3.9%) | 174 | 45.2 | 75 | 20.8 | 86 | 24.9 | 144 | 38.1 |
| Medium-low (4-7.9%) | 111 | 61.2 | 65 | 33.3 | 61 | 30.7 | 108 | 53.1 |
| Medium-high (8-11.9%) | 36 | 114.6 | 24 | 68.5 | 22 | 59.4 | 31 | 94.7 |
| High (>12%) | 43 | 109.3 | 36 | 83.2 | 44 | 108.3 | 44 | 110.4 |
| Median income |  |  |  |  |  |  |  |  |
| High (>$75,000) | 80 | 34.5 | 31 | 13.9 | 43 | 20.2 | 64 | 27.9 |
| Medium-high ($50,000-$74,999) | 119 | 48.3 | 58 | 24.2 | 66 | 28.0 | 113 | 46.6 |
| Medium-low ($25,000-$49,999) | 150 | 106.9 | 96 | 59.1 | 91 | 55.5 | 126 | 85.0 |
| Low ($0-24,999) | 15 | 83.1 | 15 | 82.7 | 13 | 72.1 | 14 | 77.0 |

.

^a^Age-adjusted incidence by three age groups: 18-49 years, 50-64 years and >65 years, standardized to the 2010 New Haven County, Connecticut, adult population.

^b^Poverty defined as the percentage of persons living below the federal poverty level in a census tract per the 2006-2010 American Community Survey.^12^

* p<0.01 by chi-square test for trend for each category in the column each year
